# Effect of the screw type (S2-alar-iliac and iliac), screw length, and screw head angle on the risk of screw and adjacent bone failures after a spinopelvic fixation technique: A finite element analysis

**DOI:** 10.1371/journal.pone.0201801

**Published:** 2018-08-16

**Authors:** Jong Ki Shin, Beop-Yong Lim, Tae Sik Goh, Seung Min Son, Hyung-Sik Kim, Jung Sub Lee, Chi-Seung Lee

**Affiliations:** 1 Department of Orthopaedic Surgery and Biomedical Research Institute, Pusan National University Hospital, Busan, Republic of Korea; 2 Department of Orthopaedic Surgery, Myung Eun Hospital, Busan, Republic of Korea; 3 Biomedical Research Institute, Pusan National University Hospital, Busan, Republic of Korea; 4 Department of Orthopaedic Surgery and Biomedical Research Institute, Pusan National University Yangsan Hospital, Yangsan, Republic of Korea; 5 School of Medicine, Pusan National University, Busan, Republic of Korea; University of Zaragoza, SPAIN

## Abstract

**Purpose:**

Spinopelvic fixations involving the S2-alar-iliac (S2AI) and iliac screws are commonly used in various spinal fusion surgeries. This study aimed to compare the biomechanical characteristics, specifically the risk of screw and adjacent bone failures of S2AI screw fixation with those of iliac screw fixation using a finite element analysis (FEA).

**Methods:**

A three-dimensional finite element (FE) model of a healthy spinopelvis was generated. The pedicle screws were placed on the L3-S1 with three different lengths of the S2AI and iliac screws (60 mm, 75 mm, and 90 mm). In particular, two types of the S2AI screw, 15°- and 30°-angled polyaxial screw, were adopted. Physiological loads, such as a combination of compression, torsion, and flexion/extension loads, were applied to the spinopelvic FE model, and the stress distribution as well as the maximum von Mises equivalent stress values were calculated.

**Results:**

For the iliac screw, the highest stress on the screw was observed with the 75-mm screw, rather than the 60-mm screw. The bones around the iliac screw indicated that the maximum equivalent stress decreased as the screw length increased. For the S2AI screw, the lowest stress was observed in the 90-mm screw length with a 30° head angle. The bones around the S2AI screw indicated that the lowest stress was observed in the 90-mm screw length and a 15° head angle.

**Conclusions:**

It was found that the S2AI screw, rather than the iliac screw, reduced the risk of implant failure for the spinopelvic fixation technique, and the 90-mm screw length with a 15° head angle for the S2AI screw could be biomechanically advantageous.

## Introduction

Degenerative spinal diseases are one of the most frequently reported chronic health problems affecting the adult population owing to aging. In addition, adult spinal deformities have increased owing to the growing elderly population. These spinal conditions lead to an imbalance in the structural support of the spine. With the development of surgical techniques and supporting surgical skills, spinal deformity surgeries have become more frequent. However, because of the poor bone quality of most elderly patients, implantation-related problems are frequent, especially in the lumbosacral area. Kim et al. [1] reported a pseudoarthrosis rate of 24% at the L5-S1 junction in adult scoliosis surgery. In addition, many studies have shown that long instrumentation to the sacrum without pelvic fixation is susceptible to implant failure [2–4].

To overcome the complications associated with fusions ending at S1, sacropelvic fixation has been introduced as a safe alternative [5–7]. Spinopelvic fixation with iliac screws has been used in the correction of various spinal deformities requiring long spinal fusions. Iliac screws consist of independent anchors that are placed in the ilium and connected modularly with modern spinal constructs consisting of rods with pedicle screws and hooks [8]. This technique provides powerful control of the pelvis; however, extensive subfascial dissection to expose the posterior-superior iliac spine (PSIS) during implant insertion is required and has caused complications. In addition, long-term problems have occurred associated with implant prominence related to the PSIS starting point [9, 10]. In one study, 22% of the patients required screw removal after 2 years [11], and in another study, the incidence of these problems was higher after 5 years [12].

The S2-alar-iliac (S2AI) technique, first proposed in 2007, uses a starting point in the sacral ala, midway between the S1 and S2 dorsal foramina along a line that connects the lateral edge of the two foramina [8]. This point is also in line with the S1 pedicle screw starting point. The S2AI technique does not require the dissection of the subcutaneous tissue over the iliac crest or the sacral paraspinous muscle, as is required for iliac screws that start at the PSIS. Decreased implant prominence is one of the advantages of this technique, as the starting point is approximately 15 mm deeper than that used for the entry to the PSIS [13, 14]. This technique allows a single rod to be used without the need for cumbersome connectors and has the potential to minimize the complexity of the procedure [15]. Moreover, S2AI screws have fewer unplanned reoperations than iliac bolts for instrumentation-related complications, wound infections, and instrumentation removal owing to pain [16]. However, one recent study reported a high rate of mechanical failure of S2AI screws in an early/midterm follow-up [17]. The failure rate of the S2AI screws was 35% compared to 12% for the iliac screws with lateral connectors.

As previously mentioned, many studies have focused on the anatomy, surgical technique, and risk evaluation during iliac and S2AI screw fixations. However, there are few studies that have performed a biomechanical assessment and comparative study of these two fixation types. Few studies have evaluated the material internal stress in the screw and the bone around the screw, under various fixations and spine motions. To evaluate the biomechanical and physical factors, such as the von Mises equivalent stress, cadaver or computational approaches to the analysis are required.

The finite element method (FEM) of computational analysis is widely used in the study of many biological systems, especially the musculoskeletal system. Through the FEM, many complex geometrical and material properties of the biological system can be effectively evaluated, and many physical variables, such as stress, strain, damage, and fracture, can be quantitatively analyzed.

In a few studies, the biomechanical ability and stability of the spinopelvic system have been numerically investigated. Garcia et al. [18] carried out a simulation study to analyze the functional performance of the pelvis and the stability of different types of fixations for several kinds of fractures. Zhao et al. [19] produced an FE model of a Tile C pelvic ring injury and compared the stability of seven types of models that were fixed using normal and lengthened sacroiliac screws for the treatment of bilateral vertical sacral fractures. Bruna-Rosso et al. [20] evaluated the biomechanical features of stable sacroiliac joint (SIJ) fixation in the physiological condition using a detailed FE model. The pre-instrumented and post-instrumented SIJ mobilities were compared using different implant configurations.

As previously mentioned, many computational studies have focused on the spinopelvic fixation analysis. However, there is no comparative study regarding the iliac and S2AI screw fixation technique. Hence, in this study, the biomechanical characteristics of the iliac and S2AI screw fixed spinopelvic system under various implant configurations were computationally evaluated, and the simulation results, such as the equivalent stress values in the surrounding bone and screw, were investigated. Based on the calculated results, an optimal implant condition was proposed.

## Materials and methods

This research was approved by the Institutional Review Board (IRB) of the Pusan National University Hospital (PNUH). The reference number is PNUH-IRB-E-2016068. An informed consent statement was signed after receiving oral description of the simulation prior to the start of simulation.

The software programs Mimics 19.0 (Materialise, Belgium), SolidWorks 2016 (Dassault Systèmes, USA), and ANSYS 16.1 (ANSYS Inc., USA) were used. A computed tomography (CT) scanner (GE, USA) was used to collect raw data in the digital imaging and communication in medicine (DICOM) format with a scan slice of 0.75 mm.

### FE models for spinopelvis and implants

An FE model of the spinopelvis (L3-Pelvis), which included three vertebrae, three discs, and a pelvis, was reconstructed. The geometrical specifications of the spinopelvis were obtained from 64 spiral CT images of a 27-year-old healthy male without a history of spine injury and osteoporosis or radiographic evidence of degeneration. The patient underwent a CT examination for a health checkup at our hospital, and the CT images were used with the patient’s consent. The date of patient recruitment for imaging was August 15, 2016, and the time period for this research was June 28, 2016 to May 31, 2017.

The CT images were scanned and imported into Mimics 19.0 to construct the three-dimensional (3D) surface of the spinopelvis. To avoid unexpected stress concentration, the surface of the spinopelvis was smoothed. The FE model was generated using the ANSYS meshing tool. A 10-node tetrahedral solid element was adopted to express the cancellous bone, and an 8-node hexahedral element with a 1-mm thickness was used to represent the cortical bone, surrounding the cancellous bone. To describe the intervertebral disc in the FE model, the nucleus pulposus and annulus fibrosus were adopted using an 8-node hexahedral solid element. The model without implants had a total of 223833 elements and 341569 nodes.

**Fig 1:**
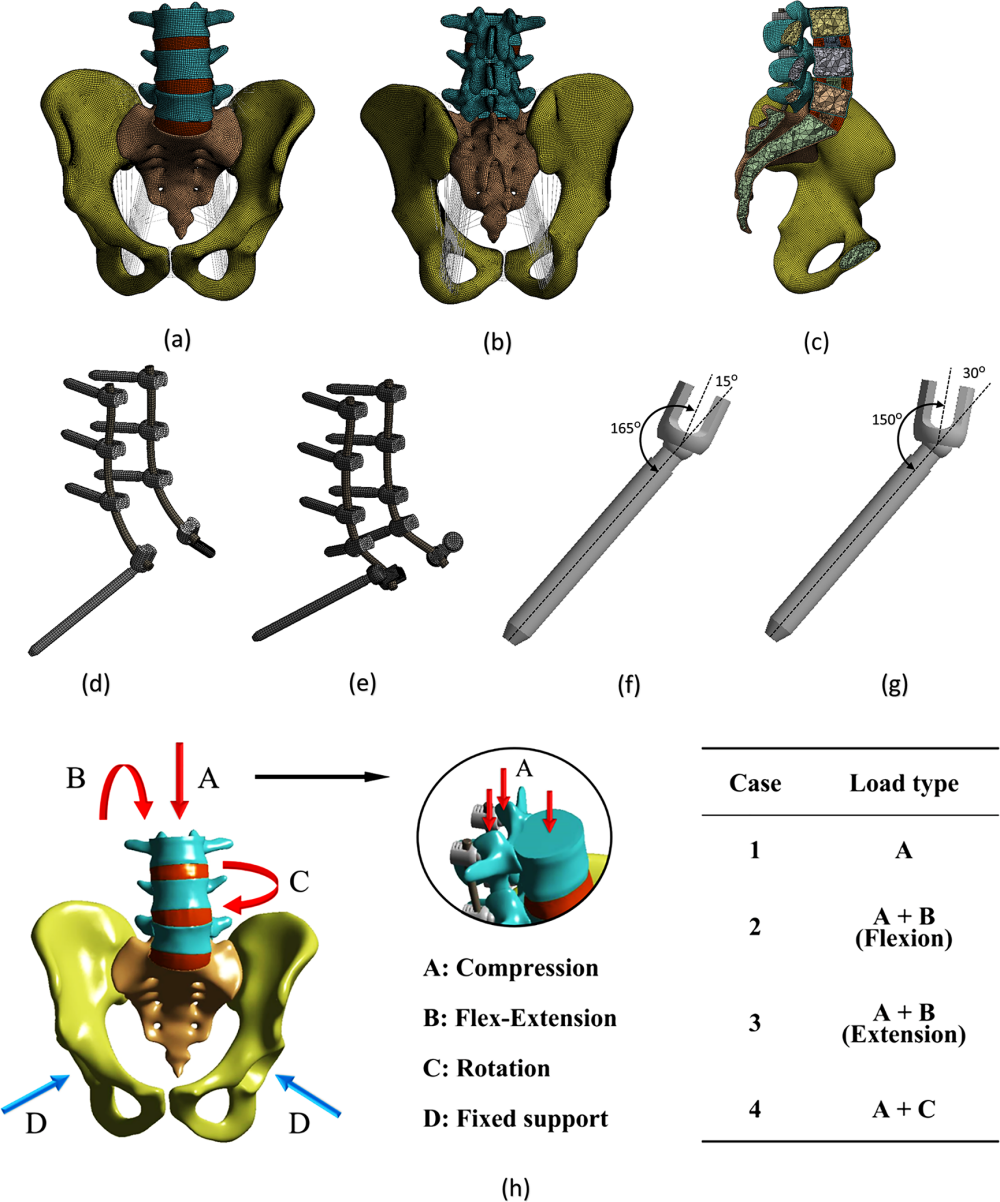
Finite element model and loading/boundary condition of the spinopelvis and implant. Image (a) shows the anterior view with ligaments, and (b) shows the posterior view with ligaments. Image (c) shows the sagittal plane view, and (d) shows the S2AI screw. Image (e) shows the iliac screw, and (f) shows the 15° head angle. Image (g) shows the 30° head angle, and (h) shows the loading/boundary condition.

Owing to their important roles in pelvic biomechanics, the anterior/posterior longitudinal, interspinous, sacroiliac, sacrospinous, sacrotuberous, and pubic ligaments were incorporated and modeled as the spring elements. The attachment points were ascertained by mimicking the anatomy as closely as possible. The final FE model of the normal spinopelvis is shown in Fig 1(A), 1(B) and 1(C).

This 3D geometry of the spinopelvic model was then exported into 2016 SolidWorks software to generate the fixation models. The polyaxial pedicle screw instrumentation (7.0 × 35 mm) was placed bilaterally from L3 to S1, and two different screws were adopted for spinopelvic fixation: iliac and S2AI screws. The diameters of the iliac and S2AI screws were 7.0 mm each, and the length of the three screws varied: 60 mm, 75 mm, and 90 mm. Two types of the S2AI screw, 15°- and 30°-angled polyaxial screws, were used.

The iliac and S2AI screws were implanted into the spinopelvic model using the standard surgical technique. The iliac screws were inserted from the PSIS to the anterior-inferior iliac spine (AIIS) at each ilium, and the lateral connectors were placed in the iliac screws. The S2AI screws were placed 1-mm inferior and 1-mm lateral to the S1 dorsal foramen. Angulation of the screw was directed just above the sciatic notch in the coronal plane.

The threads of the screws were eliminated to simplify the FE models. Based on the simplified model, the computational time as well as the analysis risk owing to an unrealistic high stress could be reduced, and a smooth transfer of stress between the screw and bone could be realized [21]. The element type of the implant was the 8-node hexahedral solid element, and the number of elements for the implants was 42139 for the iliac-screw construct and 24199 for the S2AI-screw construct. The final FE model of the implant with the iliac and S2AI screws and two types of head angles of the S2AI screw are shown in Fig 1(D), 1(E) and 1(F).

The material properties of the bones, intervertebral disc, implants, and various ligaments are listed in Tables 1 and 2. The material of the implant was Ti-6Al-4V.

**Table 1:** Material properties of the tissues and implants.

	Young's modulus (MPa)	Poisson's ratio	Element type	Reference
**Cancellous bone**	150	0.2	10-node tetrahedral solid element	[19]
**Cortical bone**	18,000	0.3	8-node hexahedral solid element	[19]
**Nucleus pulposus**	2	0.45	8-node hexahedral solid element	[22]
**Annulus fibrosus**	8	0.49	8-node hexahedral solid element	[22]
**Implant** **(Ti-6Al-4V)**	114,000	0.3	8-node hexahedral solid element	[19]

**Table 2:** Material properties of the lumbar and pelvic ligaments.

Ligament	K (N/m)	Number of springs	Reference
**Anterior longitudinal**	23.75	10	[23]
**Posterior longitudinal**	26.15	5	[23]
**Interspinous**	9.8	10	[23]
**Iliolumbar**	1000	10	[24]
**Anterior sacroiliac**	700	25	[19]
**Posterior sacroiliac**	1400	18	[19]
**Sacrospinous**	1400	10	[19]
**Sacrotuberous**	1500	15	[19]
**Superior pubic**	500	22	[19]
**Arcuate pubic**	500	22	[19]

**Fig 2:**
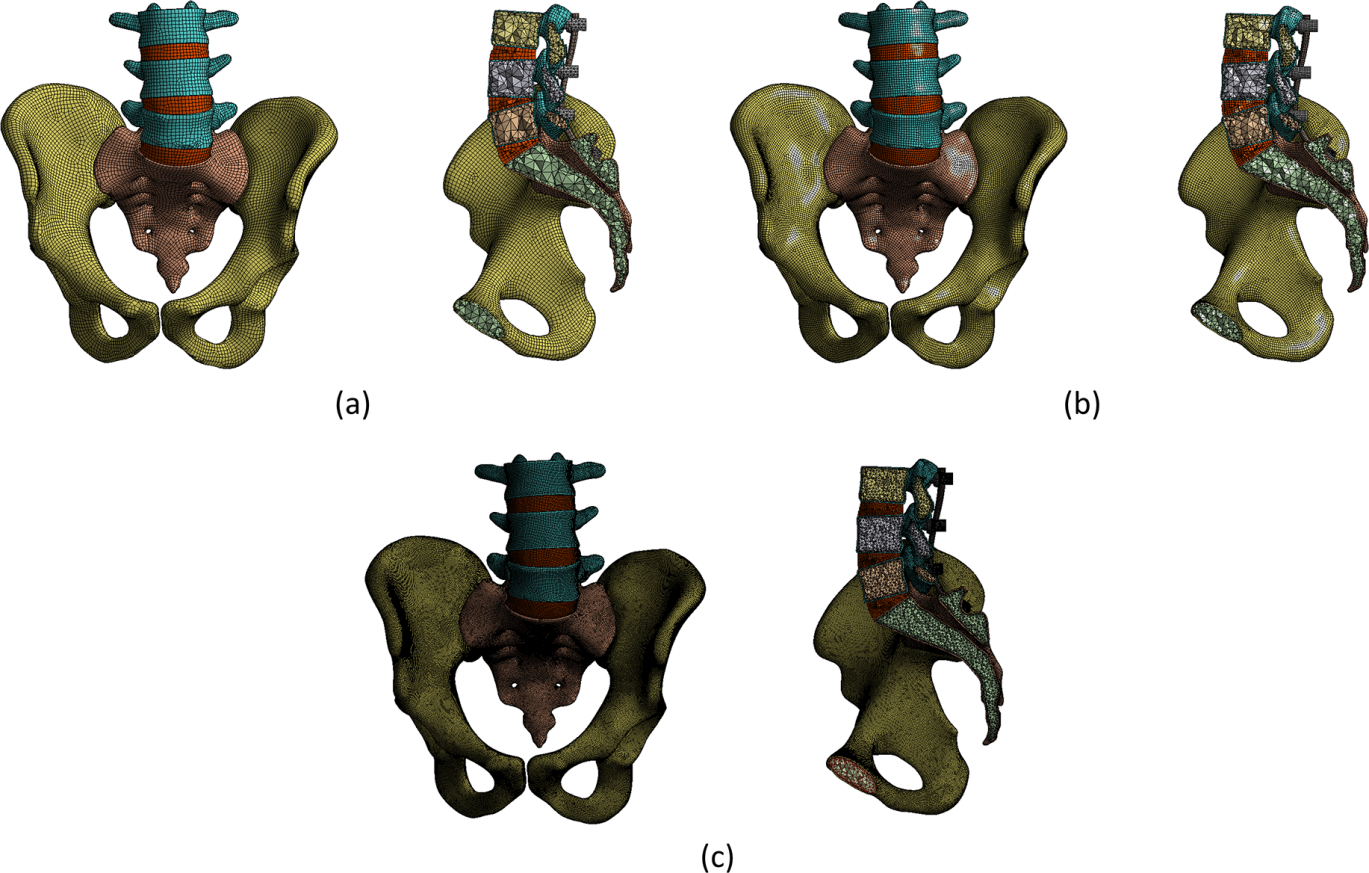
Classification according to the mesh densities to evaluate the sensitivity of the elements. Image (a) shows the coarse mesh, and (b) shows the medium mesh. Image (c) shows the fine mesh.

This study also evaluated the sensitivity of the elements that affected the accuracy of the results prior to the regular FE analysis (FEA). The coarse, medium, and fine densities of three representative meshes were selected to determine the number of elements, as shown in Fig 2.

An analysis was performed using the three densities, and the results converged from the medium- to fine-mesh densities. Table 3 lists the number of elements, number of nodes, computational time, and equivalent stress on the screw/bone for each density. For the fine mesh, the convergence and accuracy were increased; however, its use was not practical because of the increased computational time. Therefore, the medium mesh with a high degree of convergence was selected for the analysis.

**Table 3:** Analysis of the state and influence on the interpretation by densities of the mesh.

	Coarse	Medium	Fine
**Number of elements**	215,230	494,572	883,596
**Number of nodes**	372,908	878,898	1,775,278
**Approximate computational time (min)**	5	20	300
**Stress on the screw (MPa)**	64.19	74.76	75.62
**Stress on the bone (MPa)**	23.41	35.57	37.23

### FEA procedure

The ANSYS 16.1 FEA software program was used to calculate the stress distribution in each model. As listed in Tables 1 and 2, linear elastic isotropic material properties were assigned to all tissues and implants [21, 25, 26].

The bilateral acetabulum of the FE model was fixed. The interface condition between the screw and bone plays a vital role in determining the stress distribution [25]. Accordingly, a surface-to-surface contact element was adopted to simulate the contact interfacial characteristics between the screw and bone. In this study, however, a large amount of computational time was required to calculate the stress distribution in the bone-screw interface owing to the large number of contact interfaces. Moreover, there was a convergence problem with the contact condition during the calculation since the number of contact nodes/elements was large. Therefore, the contact condition was postulated as a rigid alternative. The implants were locked to the bone to describe the fixation [26]. In addition, the contact behavior between the screw and spinal rod interfaces was set as a rigid bond. For the behavior of the spinopelvis, the facet joints or SIJs were materialized by inserting a contact condition between the abutted bones. The facet joints were assumed to be frictionless contact because it was routinely removed during surgery [23]. The SIJs were considered to be a bonded contact because it was outside the main concern of this study [21].

A pure moment of 10 Nm combined with a pre-compressive load of 700 N was applied to the top surface of L3 and the superior articular processes. 60% (500 N) and 40% (100 N) of the total load were separately applied to the upper vertebral body and superior articular processes, respectively, as the facet joints can commonly carry 10% to 40% of the compressive load of the total force subjected to the vertebrae [27–29].

Through the combination of the moment and compression, four types of loads, compression, flexion, extension, and rotation, were generated and adopted to the FE model. The loading and boundary conditions are shown in Fig 1(G).

### Validation of the FE model

**Fig 3:**
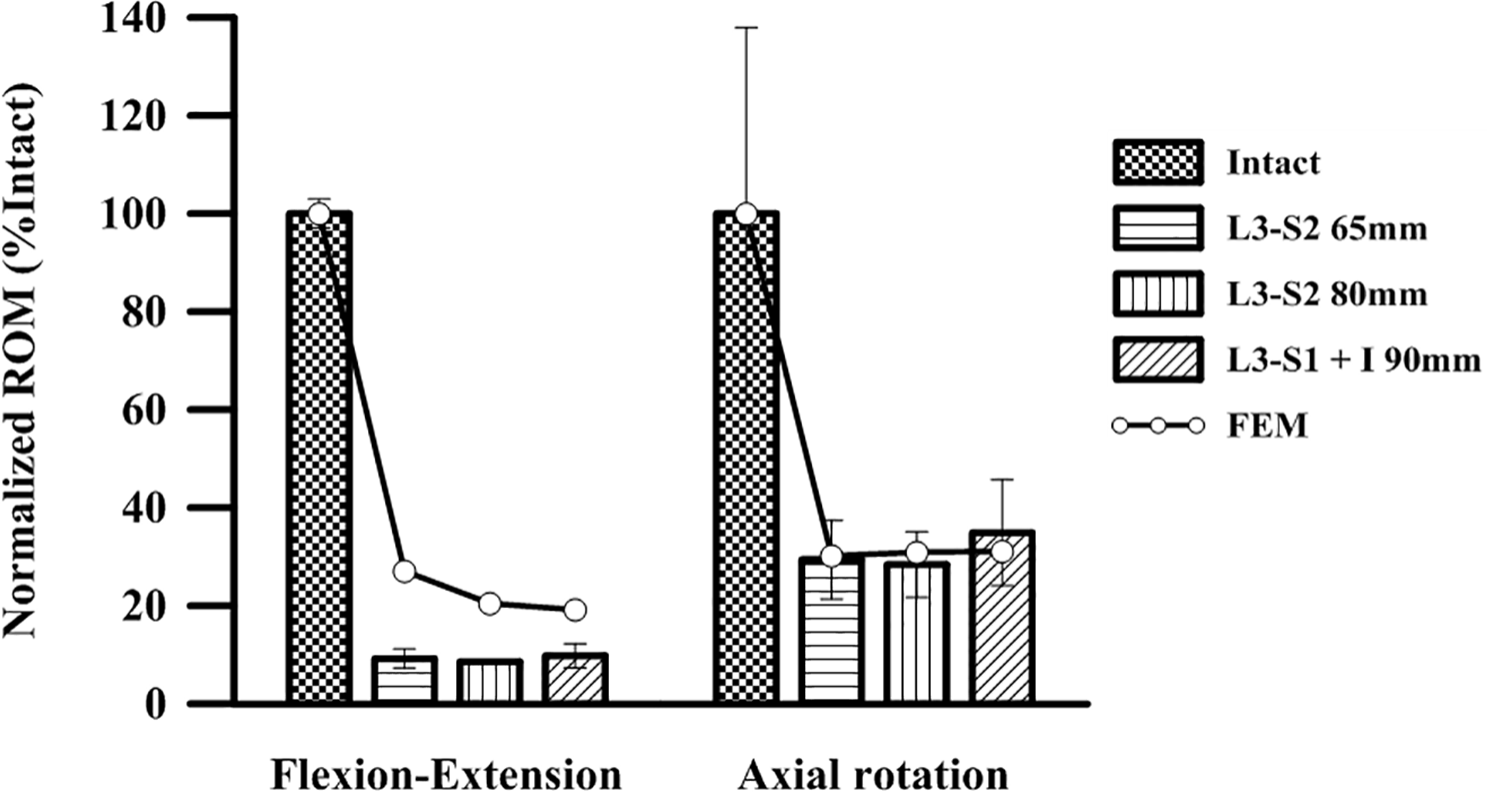
Comparison of the experimental data from the literature with the simulation results of this study.

The proposed FE model was validated by simulating the experimental cadaveric study of O’Brien et al. [30]. The normalized range of motion (ROM) test at the L3-pelvis in a cadaveric study was simulated. Then, the three types of screws (65-mm and 80-mm S2AI screws and a 90-mm iliac screw) and two types of the mechanical behaviors (flexion-extension and axial rotation) were simulated with the FE model. The simulation results were compared with the experimental results in the literature, as shown in Fig 3.

As shown in Fig 3, the simulation results in the axial rotation case coincided well with the experimental results. However, some differences in the normalized ROM values were observed between the simulation and experimental results in the flexion-extension case. The error ranges were approximately 0.5–3.0% in the flexion-extension case and 6.7–38.0% in the axial rotation case. This was because of the anatomical shape as well as the material properties of the spinopelvis for the FE model and the cadaver do not correspond. Because of the limitations of the cadaveric study of O’Brien et al., the crucial factors, such as the anatomical information of the spinopelvis, material properties of the hard/soft tissues, and implementation position of the pedicle screw, as well as the spinal rod, could not be evaluated prior to the simulation. Despite this, the proposed FE model and FEA procedure in this study might be reasonable for the computational biomechanical investigation of the spinopelvic fixation technique.

## Results

### FEA results

**Fig 4:**
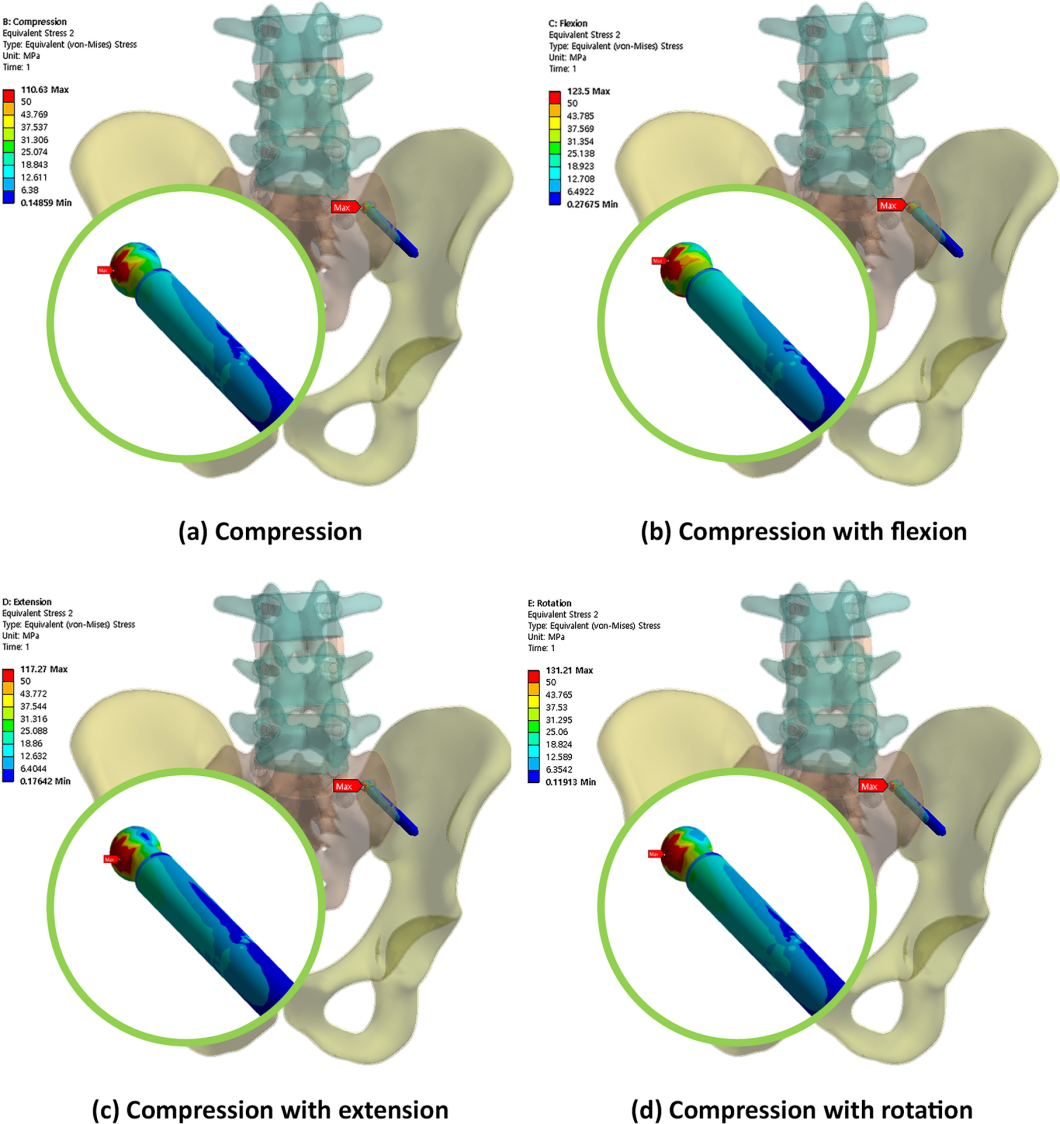
von Mises equivalent stress distribution of the screw in the spinopelvic model with the iliac screw under various loading conditions.

**Fig 5:**
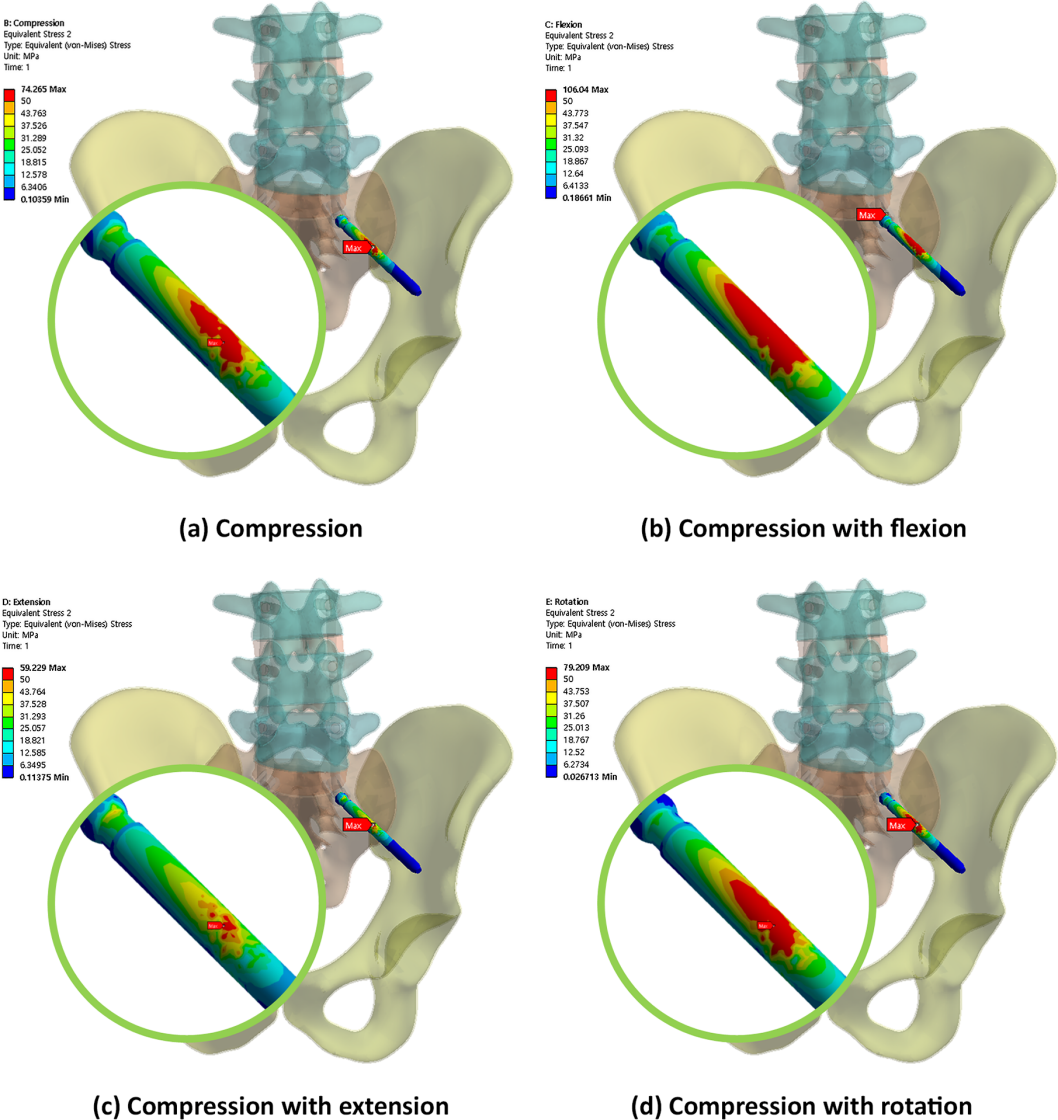
von Mises equivalent stress distribution of the screw in the spinopelvic model with the S2AI screw (75-mm screw length and 15° head angle) under various loading conditions.

**Fig 6:**
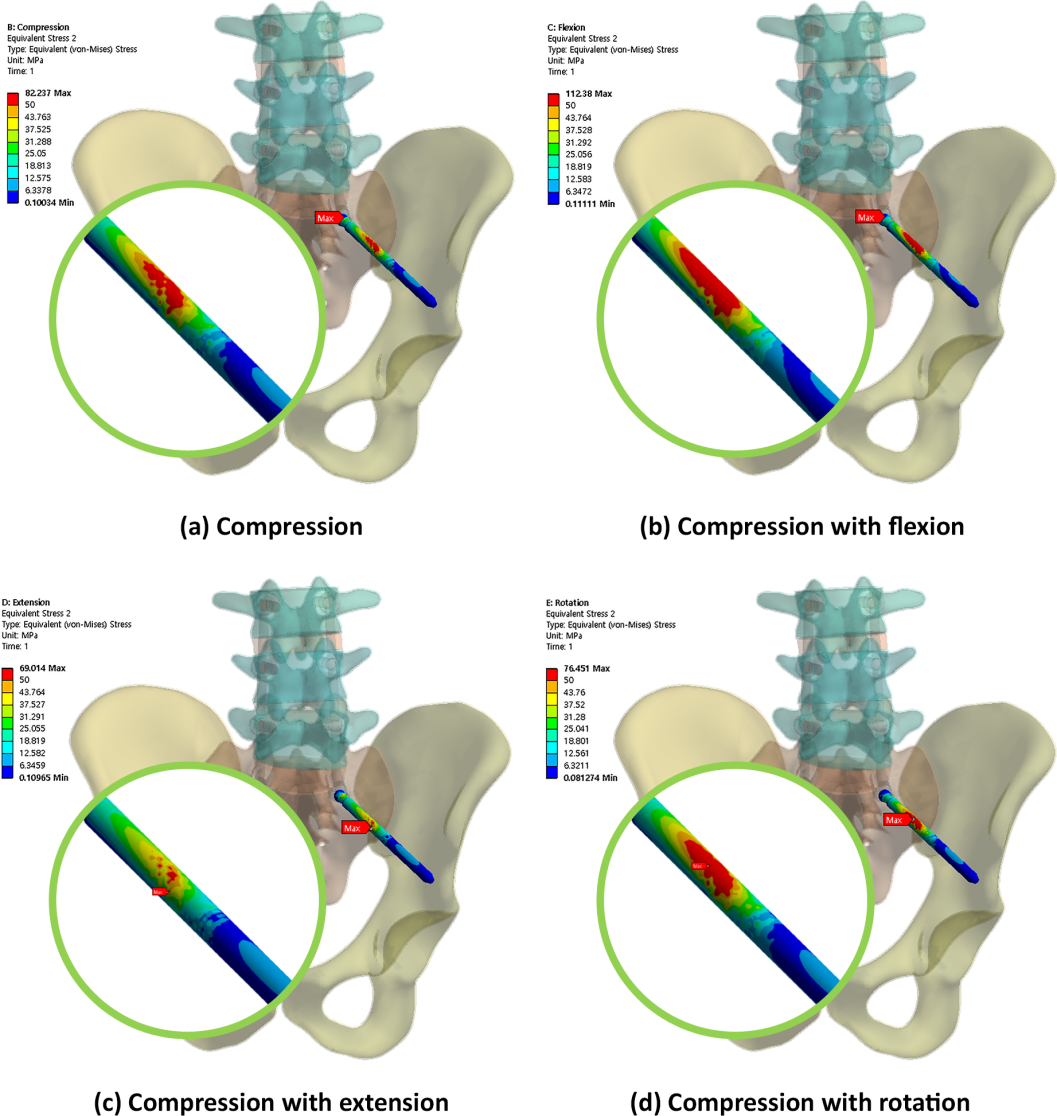
von Mises equivalent stress distribution of the screw in the spinopelvic model with the S2AI screw (90-mm screw length and 30° head angle) under various loading conditions.

Figs 4, 5 and 6 show the von Mises equivalent stress distribution of the screw in the spinopelvic model with the iliac and S2AI screws under various loading conditions. Fig 4 shows the iliac screw with a 60-mm length, and Figs 5 and 6 show the S2AI screws with a 75-mm length and a 15° head angle and a 90-mm length with a 30° head angle, respectively.

**Fig 7:**
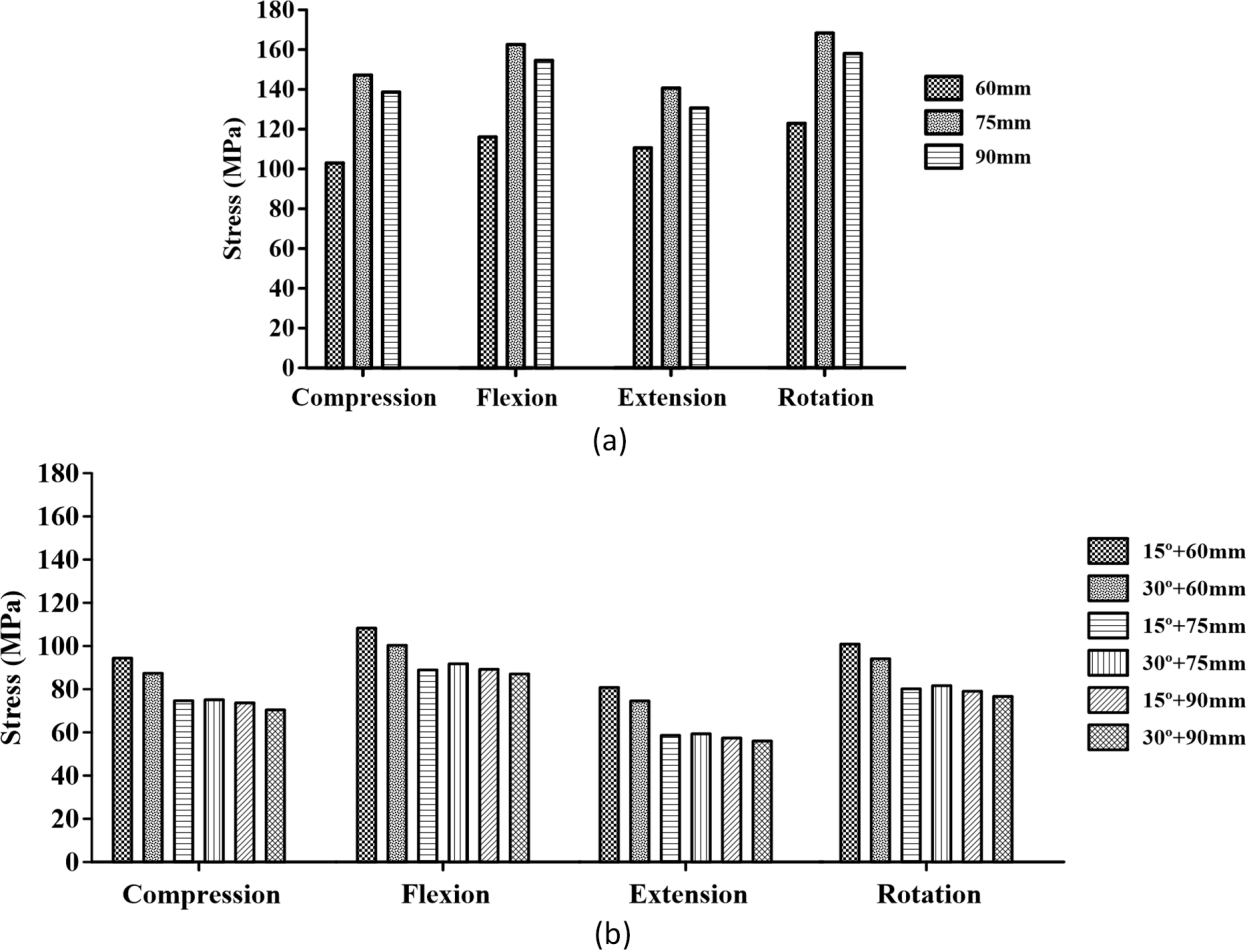
Maximum equivalent stress in the two types of screws. Image (a) shows the iliac screw, and image (b) shows the S2AI screw.

In addition, Fig 7 shows the maximum equivalent stress in the screw with respect to the screw type (iliac and S2AI screws), screw length (60, 75, and 90 mm), and head angle of the S2AI screw (15° and 30°) under four types of loading conditions.

**Fig 8:**
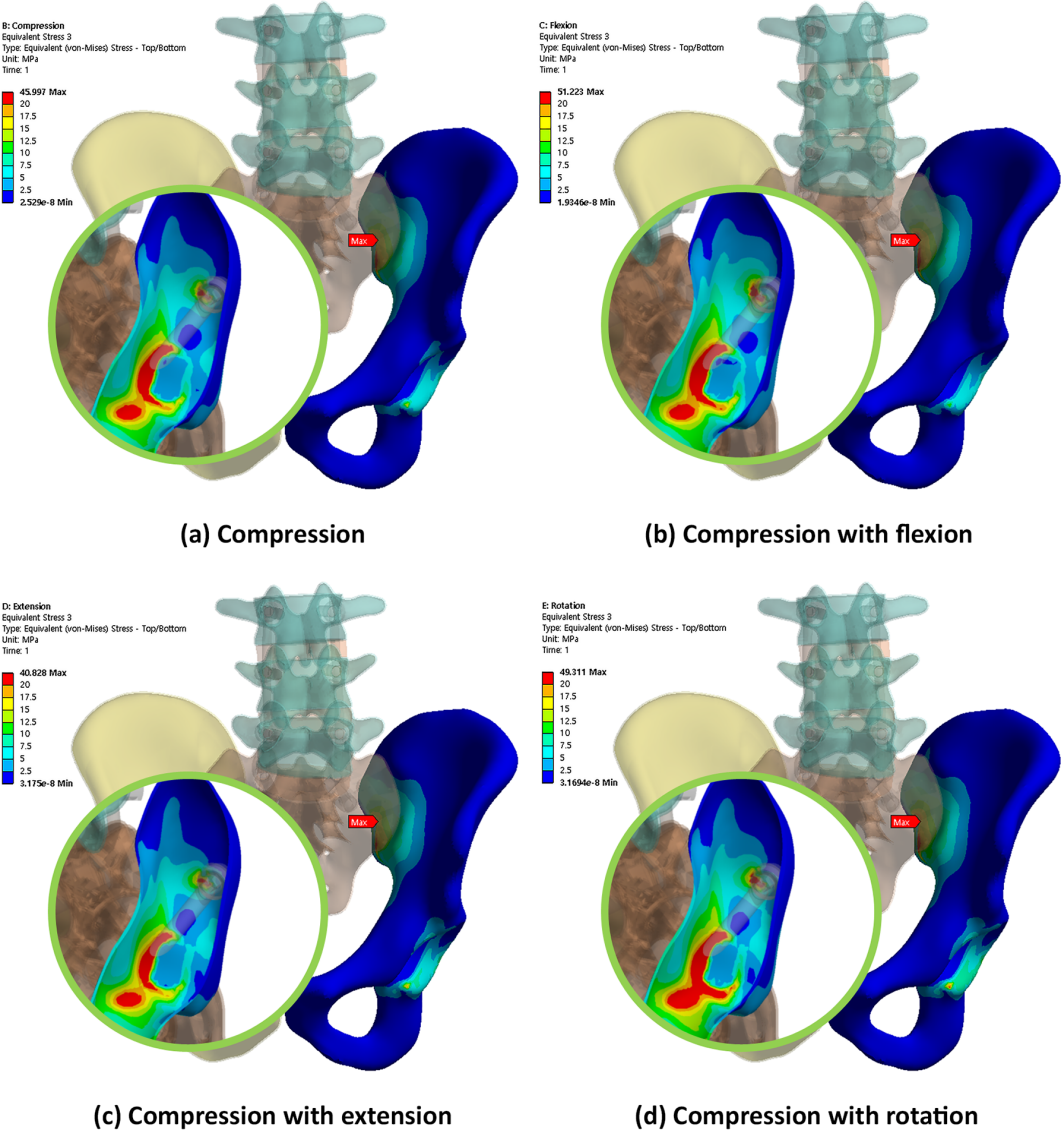
von Mises equivalent stress distribution of the bone around the iliac screw under various loading conditions.

**Fig 9:**
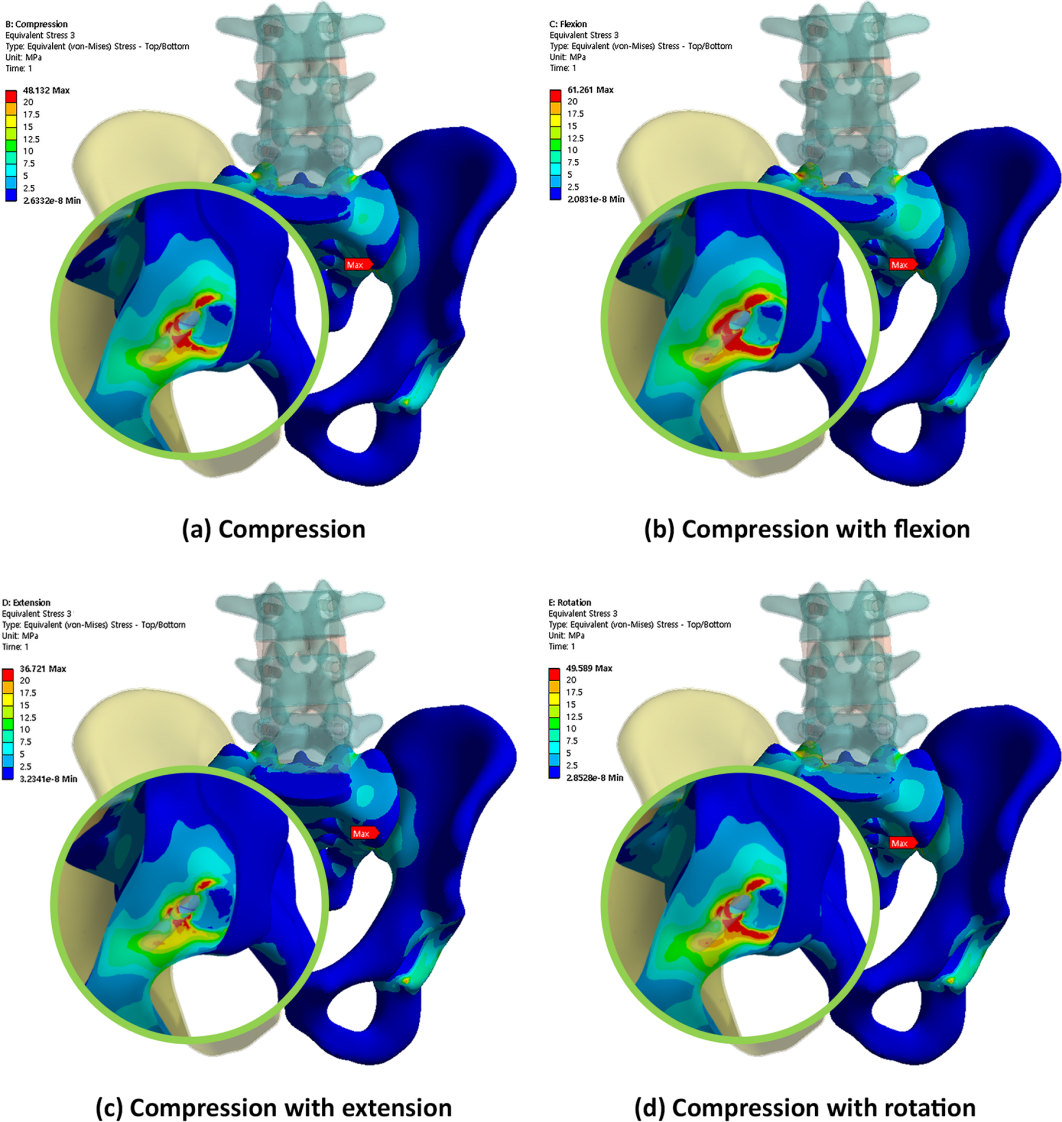
von Mises equivalent stress distribution of the bone around the S2AI screw (75-mm screw length and 15° head angle) under various loading conditions.

**Fig 10:**
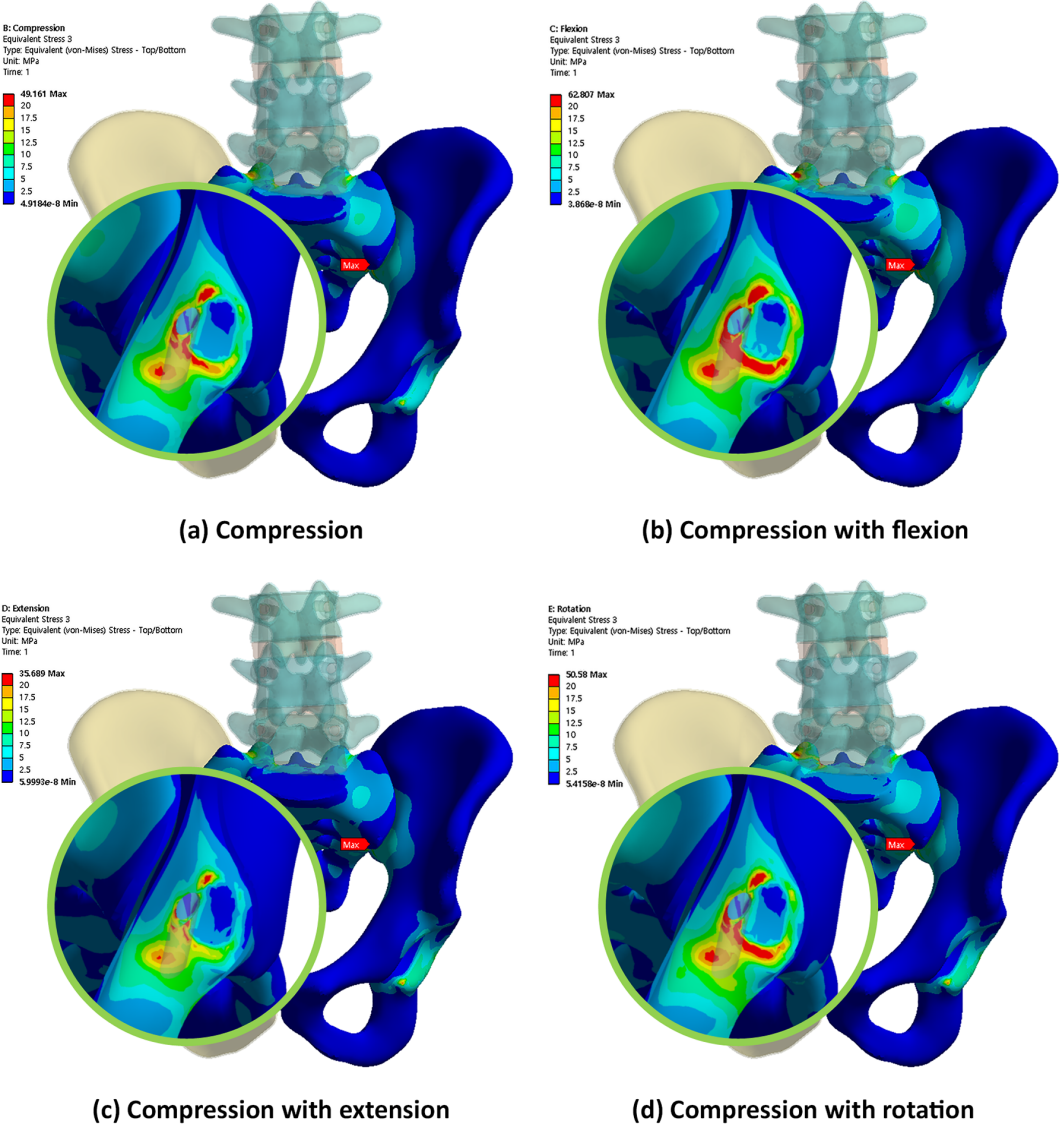
von Mises equivalent stress distribution of the bone around the S2AI screw (90-mm screw length and 30° head angle) under various loading conditions.

Conversely, Figs 8, 9 and 10 show the equivalent stress distribution of the bone around the iliac and S2AI screws under various loading conditions, respectively. Fig 8 shows the bone around the iliac screw with a 60-mm length, and Figs 9 and 10 show the bone around the S2AI screws with a 75-mm length and a 15° head angle and a 90-mm length with a 30° head angle, respectively.

**Fig 11:**
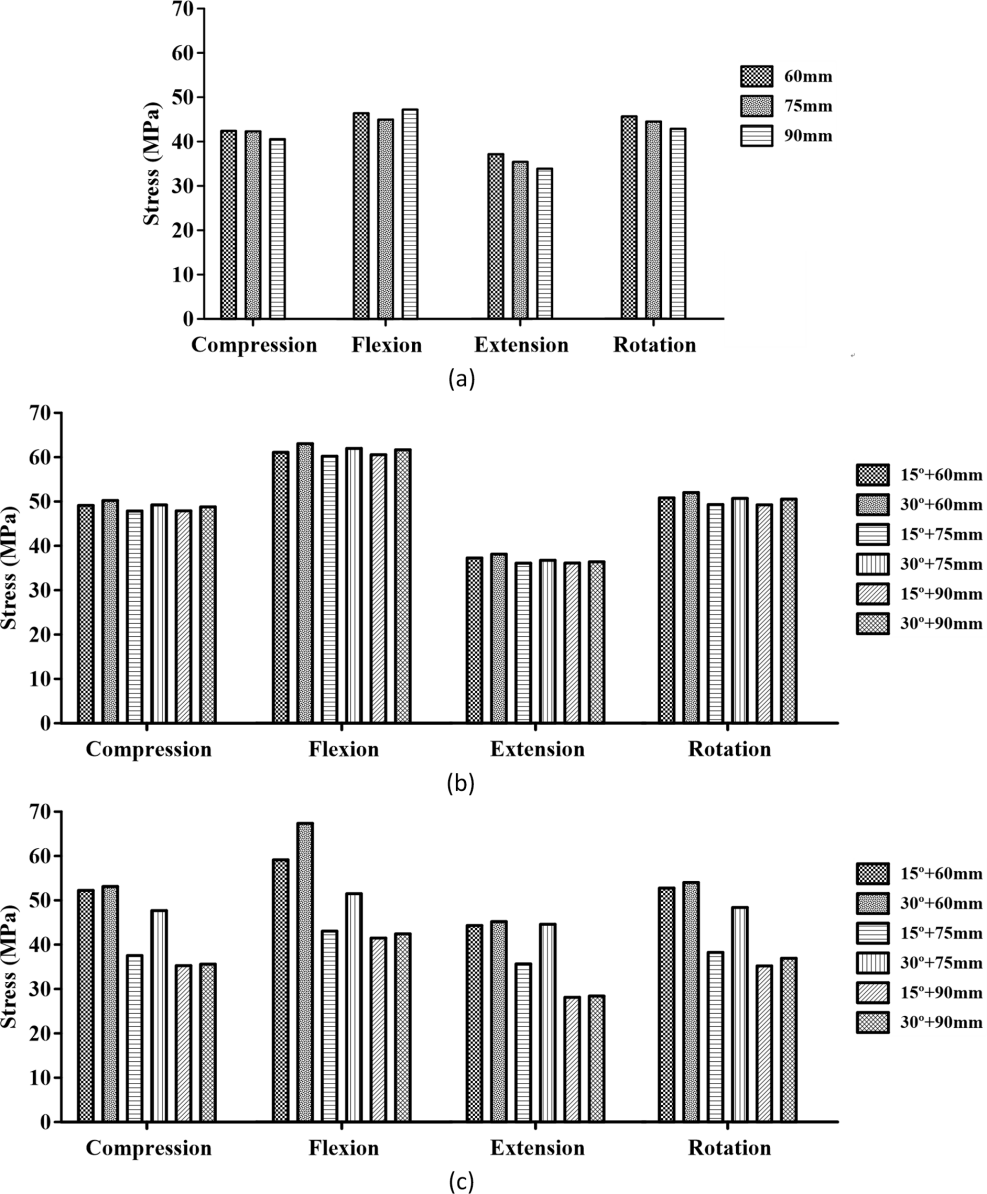
Maximum equivalent stress in the adjacent bone of the screws. Image (a) shows the iliac screw, and image (b) shows the sacrum with the S2AI screw. Image (c) shows the ilium with the S2AI screw.

Moreover, Fig 11 shows the comparison results of the maximum equivalent stress in the bone around the screws regarding the screw type, screw length, and head angle of the S2AI screw under four types of loading conditions. Since the S2AI screw penetrates the SIJ, the stress in the sacrum, as well as that in the ilium, were addressed in the graph simultaneously.

All simulation results are listed quantitatively in Table 3. In this table, the maximum equivalent stress on each screw and surrounding bone are listed. In addition, the increase/decrease in the quantity of the stress in all cases relative to those in the reference cases were identified. In this study, the reference cases were the 60-mm screw length and the 15° head angle in the S2AI screw and the 60-mm screw length in the iliac screw.

The simulation results according to the screw length, head angle, and screw type are listed below. For the iliac screw, the maximum equivalent stress decreased in the order of 75-mm, 90-mm, and 60-mm screw lengths in all loading conditions, as shown in Fig 6 and listed in Tables 4–7. The highest stress was observed in the 75-mm screw length and not in that of the 60-mm screw length. The bones around the iliac screw showed that the maximum equivalent stress decreased in the screws in the order of 60-mm, 75-mm, and 90-mm screw lengths in all loading conditions, except compression with the flexion case, as shown in Fig 11 and listed in Tables 4 to 7.

**Table 4:** Maximum von Mises equivalent stress values of the screws and the bone around the screw under compression load.

Length of screw (mm)		60		75		90	
Angle (Degree)		15	30	15	30	15	30
**S2AI screw**	von-Mises Stress (MPa)	94.70	87.60	73.30	73.71	72.85	70.39
	Percent of change (%)	0.00	-7.50	-22.60	-22.16	-23.07	-25.67
**Bone** **(Ilium)**	von-Mises Stress (MPa)	52.46	52.75	40.06	48.28	35.24	35.65
	Percent of change (%)	0.00	0.57	-23.64	-7.97	-32.83	-32.04
**Bone** **(Sacrum)**	von-Mises Stress (MPa)	49.50	50.65	47.91	49.20	48.28	49.12
	Percent of change (%)	0.00	2.32	-3.21	-0.61	-2.46	-0.68
**Iliac screw**	von-Mises Stress (MPa)	110.63		156.40		147.36	
	Percent of change (%)	0.00		41.37		33.20	
**Bone** **(Ilium)**	von-Mises Stress (MPa)	46.00		45.77		45.44	
	Percent of change (%)	0.00		-0.50		-1.22	

**Table 5:** Maximum von Mises equivalent stress values of the screws and the bone around the screw under compression and flexion loads.

Length of screw (mm)		60		75		90	
Angle (Degree)		15	30	15	30	15	30
**S2AI screw**	von-Mises Stress (MPa)	109.75	101.16	91.30	92.52	90.10	86.70
	Percent of change (%)	0.00	-7.83	-16.81	-15.70	-17.90	-21.00
**Bone** **(Ilium)**	von-Mises Stress (MPa)	60.82	61.94	43.21	52.09	43.03	42.89
	Percent of change (%)	0.00	1.84	-28.95	-14.35	-29.25	-29.48
**Bone** **(Sacrum)**	von-Mises Stress (MPa)	62.39	63.12	61.00	63.21	61.53	62.70
	Percent of change (%)	0.00	1.17	-2.23	1.31	-2.22	0.50
**Iliac screw**	von-Mises Stress (MPa)	123.5		172.28		163.76	
	Percent of change (%)	0.00		39.50		32.60	
**Bone** **(Ilium)**	von-Mises Stress (MPa)	51.22		50.71		50.46	
	Percent of change (%)	0.00		-1.00		-1.48	

**Table 6:** Maximum von Mises equivalent stress values of the screws and the bone around the screw under compression and extension loads.

Length of screw (mm)		60		75		90	
Angle (Degree)		15	30	15	30	15	30
**S2AI screw**	von-Mises Stress (MPa)	79.98	73.73	56.11	57.36	55.66	54.18
	Percent of change (%)	0.00	-7.81	-29.84	-28.28	-30.41	-32.26
**Bone** **(Ilium)**	von-Mises Stress (MPa)	44.27	44.91	36.41	44.66	27.84	28.12
	Percent of change (%)	0.00	1.45	-17.75	0.88	-37.11	-36.48
**Bone** **(Sacrum)**	von-Mises Stress (MPa)	36.71	37.30	35.21	36.02	35.00	35.61
	Percent of change (%)	0.00	1.61	-4.09	-0.97	-4.66	-3.00
**Iliac screw**	von-Mises Stress (MPa)	117.27		148.85		138.42	
	Percent of change (%)	0.00		26.93		18.04	
**Bone** **(Ilium)**	von-Mises Stress (MPa)	40.83		40.49		40.39	
	Percent of change (%)	0.00		-0.83		-1.08	

**Table 7:** Maximum von Mises equivalent stress values of the screws and the bone around the screw under compression and rotation loads.

Length of screw (mm)		60		75		90	
Angle (Degree)		15	30	15	30	15	30
**S2AI screw**	von-Mises Stress (MPa)	101.11	94.28	79.21	80.99	78.12	76.45
	Percent of change (%)	0.00	-6.76	-21.65	-19.90	-22.74	-24.39
**Bone** **(Ilium)**	von-Mises Stress (MPa)	53.27	54.44	40.50	48.82	36.52	37.08
	Percent of change (%)	0.00	2.20	-23.97	-8.35	-31.44	-30.39
**Bone** **(Sacrum)**	von-Mises Stress (MPa)	50.91	52.02	49.33	50.91	49.75	50.53
	Percent of change (%)	0.00	2.18	-3.10	0.00	-2.28	-0.75
**Iliac screw**	von-Mises Stress (MPa)	131.21		178.29		167.49	
	Percent of change (%)	0.00		35.88		27.65	
**Bone** **(Ilium)**	von-Mises Stress (MPa)	49.31		49.03		48.77	
	Percent of change (%)	0.00		-0.57		-1.10	

Conversely, for the S2AI screw, the highest and lowest maximum equivalent stresses occurred in the 60-mm screw length with a 15° head angle and the 90-mm screw length with a 30° head angle, respectively, in all loading conditions, as shown in Fig 7 and listed in Tables 4 to 7. The maximum equivalent stress on the sacrum and ilium was found in the S2AI screw, as shown in Fig 11 and listed in Tables 4 to 7. The bones around the S2AI screw indicated that the lowest stress was observed in the 90-mm screw length with a 15° head angle on the sacrum and ilium. Although there was no large variation (or percent of change) of the stress in the sacrum, a large variation of stress was observed in the ilium.

The stress ranges of the iliac screw and surrounding iliac bone were approximately 111–178 MPa and 40–51 MPa, respectively. In addition, the stress ranges of the S2AI screw and surrounding sacrum/iliac bones were approximately 54–110 MPa and 28–63 MPa, respectively.

## Discussion

The aim of this study was to evaluate the biomechanical characteristics of two types of spinopelvic fixation systems under various implant configurations and loading conditions. To obtain the stress contour of spinopelvic fixation using FEM, 3D FE models for the cortical and cancellous bones and the intervertebral disc, including the nucleus pulposus and annulus fibrosus, which have a significant influence on the mechanical behavior of the spinopelvis, were fabricated. Moreover, 11 types of lumbar and pelvic ligaments were considered to accurately represent the human spinopelvic state. Two types of implants for iliac and S2AI fixation were also generated and inserted into the spinopelvic FE model. Based on a series of simulations under compression, flexion, extension, and axial rotation, the von Mises equivalent stress values in the screws and bony structures were calculated, and the simulation results were compared.

An ideal internal fixation method should provide a maximum rigidity between the bone segments and a minimum stress on the surrounding tissues for healing. Excessive stress around the fixation devices can cause gradual resorption of the surrounding bone and loosening of the screws. This is an important clinical aspect that must be considered when choosing the appropriate rigid fixation system [31]. The simulation results in this study showed that the maximum equivalent stress on the screw and bone around the screw in S2AI screw fixation was lower than that in iliac screw fixation. Thus, the S2AI screw method was a more suitable spinopelvic fixation technique than that of the iliac screw method.

According to previous studies on the stability of spinopelvic fixation, S2AI screw fixation can lead to a more reliable stability compared to iliac screw fixation owing to its longer screw length [32, 33]. Conversely, Chang et al. [13] reported that the maximum mean iliac length from the PSIS to the AIIS was 118 mm, which was longer than the maximum mean length based on the ideal trajectory for the S2AI pathway (106 mm). Recent literature indicated that iliac screw lengths chosen by surgeons, depending on the size of the pelvis, varied from approximately 50–75 mm [34]. Thus, although the S2AI trajectory allows a shorter anchor length, it still exceeds the length typically used. In one biomechanical study comparing S2AI and iliac screws, it was explained that iliac fixation is generally a cancellous bone bed, while S2AI screws have cortical purchase in the SIJ articulation and may offer additional strength despite the shorter length [30]. In their study, the 65-mm S2AI screws were not biomechanically different from the 80-mm S2AI screws or the 90-mm iliac screws. Moreover, they postulated that the quad-cortical S2AI screw placement may improve its biomechanical property.

However, in this study, the difference in the maximum equivalent stress on the screw and the bone around the screw owing to the screw length was confirmed. For the S2AI screw, as the screw length increased, the magnitude of the stress on the screw decreased, and the maximum equivalent stress value on the ilium around the screw decreased as well. There was no large variation in the stress on the sacrum based on the screw length. Conversely, for the iliac screw, there was no proportional relationship between the screw length and the maximum equivalent stress on the screw. As described in the Results section, the maximum equivalent stress occurred in the 75-mm screw length. Nevertheless, as the screw length increased, the maximum equivalent stress on the ilium decreased, except in the flexion state.

Guler et al. [17] reported that the failure rate of the S2AI screws was 35%, and that of the iliac screws was 12%, with lateral connectors. There were three cases implanted with S2AI screws, in which the polyaxial screw head disintegrated from the screw shaft in 20 patients. Compared to the monoaxial screw, the polyaxial screw is more widely used because the surgeon can easily insert the rod into the screw head owing to its degrees of freedom. In many studies comparing the biomechanical effects of monoaxial and polyaxial pedicle screw fixations, it has been concluded that the load at the bone and implant interface may decrease as the degree of freedom of the implant increases [35–39]. Fogel et al. [36] reported that the polyaxial head coupling to the screw is the first to fail and may be a protective feature of the pedicle screw, preventing pedicle screw breakage. In this study, the maximum equivalent stress values on the S2AI screw and the bone around the screw were compared while changing the screw head coupling angle to 15° and 30°. With respect to the stress, the 30° and 15° head angles for the S2AI screw were advantageous to the screw and bone, respectively, since the low stress in the material and/or structure implied a higher safety. The calculated maximum equivalent stress ranges for the S2AI screw and ilium were approximately 54–110 MPa and 28–63 MPa, respectively. Moreover, the yield stresses for Ti-6Al-4V and the cortical bone were 874 MPa and 135 MPa, respectively [40, 41]. Since the maximum equivalent stress of the screw was lower than the yield stress of the screw, the head angle of the S2AI screw should be chosen based on the bone material capacity. Therefore, the screw with a 15° head angle was more suitable for the fixation method.

For the accuracy and verification of the simulation results, it was necessary to specify the maximum von Mises equivalent stress point of each case. In the iliac screw, the maximum equivalent stresses of the screws were at the point where the screw and iliac cortical bone met, and the maximum equivalent stresses of the bones were in the iliac cortical bone at the SIJ. The maximum equivalent stresses occurred at the head portion of the screws in a few cases; however, these were beyond the scope of this study. Therefore, the point measured was the one at which the SIJ was not in contact with the head. When the S2AI screw was inserted, it was passed through the inside of the sacrum and ilium. At this time, the SIJ was penetrated through the cortical bone, and the maximum equivalent stress point was in the sacral and iliac cortical bone.

The limitations of this study are addressed below. First, the threads of the pedicle, iliac, and S2AI screws in the spinopelvic FE model were simplified to reduce the computational time and analysis error owing to the stress concentration. However, to obtain an accurate stress distribution on the screw and the bone around the screw, the threads of the screw should be considered in the FE model. Specifically, the contact condition between the bone and implant should be precisely considered during the FEA.

Second, the spinopelvic FE model in this study was asymmetrical. Accordingly, the stress distribution as well as the occurrence position of the maximum equivalent stress were not even. To acquire precise simulation results, this problem should be solved.

Third, 11 types of ligaments were modeled as a spring element, and the intervertebral disc, including the nucleus pulposus and annulus fibrosus, was simply fabricated using a solid element. In addition, the endplate of the vertebra was ignored. Since the ligaments, intervertebral disc, and endplate play a vital role in the biomechanics of the spinopelvis, it is necessary to model these tissues in detail. For example, the ligaments could be modeled as a shell/solid element, and the endplate and annulus ground substance should be considered.

Fourth, the pubic symphysis was modeled with 24 spring elements that could match the equivalent stiffness of the symphysis owing to the convenience of FE modeling [19]. However, this tissue is a cartilaginous joint that is thick and stiff. Hence, it might be possible to obtain the precise calculation results if this part was modeled as 3D solid or shell elements. Similarly, the bone-screw interface and SIJ were postulated as a bonded contact; however, this is a limitation of this study as well.

Fifth, this study required various parametric analyses to obtain highly accurate results. A wide range of parametric analyses and numerical experiments using an advanced FE model and FEA should be helped comparative analysis and improvement, provided to further the optimal surgical plan and implant configuration.

In conclusion, by comparing the biomechanical characteristics of the S2AI screw and Iliac screw, the S2AI screw showed a lower risk of implant failure than that of the iliac screw for the spinopelvic fixation technique. From analyzing the length and angle characteristics, the 90-mm screw length as well as the 15° head angle for the S2AI screw were considered biomechanically advantageous.
